# Physical activity, sedentary behavior, and risk of sepsis: a two-sample mendelian randomization study

**DOI:** 10.3389/fmed.2024.1436546

**Published:** 2024-08-19

**Authors:** Yang Zhang, Yu Rong, Jun Mao, Jin Zhang, Wenyan Xiao, Min Yang

**Affiliations:** ^1^The Second Department of Critical Care Medicine, The Second Affiliated Hospital of Anhui Medical University, Hefei, Anhui, China; ^2^Laboratory of Cardiopulmonary Resuscitation and Critical Care, The Second Affiliated Hospital of Anhui Medical University, Hefei, Anhui, China; ^3^Anqing Municipal Hospital, Anqing, Anhui, China

**Keywords:** sepsis, physical activity, sedentary behavior, risk, mendelian randomization

## Abstract

**Objective:**

This investigation aimed to explore the potential causal relationship between physical activity, sedentary behavior and the risk of sepsis.

**Methods:**

Using a two-sample Mendelian randomization approach, this study evaluated the association between physical activity (including moderate to vigorous physical activity [MVPA], vigorous physical activity [VPA], and accelerometer assessed physical activity) and sedentary behaviors (including television watching, computer use, and driving) with the risk of sepsis. This assessment was based on whole-genome association study data from the UK Biobank and the FinnGen database. Causal inferences were estimated using inverse variance-weighted, weighted median, and MR-Egger methods. Sensitivity analyses were performed using Cochran’s Q test, the MR-Egger intercept test, and the leave-one-out method.

**Results:**

The risk of sepsis was significantly inversely associated with genetically predicted MVPA (odds ratio [OR] 0.47, 95% confidence interval [CI] 0.24–0.93, *P* = 0.0296) and VPA alone (OR 0.19, 95% CI 0.04–0.87, *P* = 0.0324). Conversely, prolonged driving time showed a significant positive association with the risk of sepsis (OR 3.99, 95% CI 1.40–11.40, *P* = 0.0097).

**Conclusion:**

This study provides preliminary evidence of a causal relationship between MVPA and VPA and a reduced risk of sepsis, while prolonged sedentary behaviors such as driving are positively associated with an increased risk of sepsis. These findings provided essential scientific evidence for the development of effective sepsis prevention strategies.

## Introduction

Sepsis is an organ dysfunction resulting from a dysregulated host response to infection, which can lead to organ failure, shock, and even death in severe cases (1). Approximately 48 million people worldwide are afflicted with sepsis each year, leading to around 11 million deaths, making it a significant global public health concern due to its high mortality and morbidity rates, as well as the substantial economic burden it imposes (2). In recent years, despite a deeper understanding of the pathophysiological mechanisms of sepsis and significant advancements in hemodynamic monitoring and resuscitation strategies, the prognosis for sepsis remains suboptimal (3). Therefore, research into strategies to prevent and reduce sepsis is critical, particularly in identifying potential modifiable risk factors.

Physical activity and sedentary behavior have been extensively studied and are considered closely related to an individual’s health status as well as the occurrence of various chronic diseases (4–6). Research indicates that reducing sedentary behavior and moderately increasing physical activity can prevent the onset of numerous chronic diseases, with possible mechanisms including improved metabolic health, enhanced immune function, and reduced levels of systemic inflammation (4, 7–9). These mechanisms not only aid in preventing chronic diseases but are also closely associated with a reduced risk of sepsis. Given that physical activity and sedentary behavior are modifiable factors, it is necessary to explore the relationship between these behaviors and the risk of sepsis.

Due to limitations in cost, resources, and ethical considerations, there are currently no randomized controlled trials examining the relationship between physical activity, sedentary behavior, and the risk of sepsis. The few existing studies are observational in nature, which are susceptible to confounding factors and reverse causation, making it challenging to establish definitive causal conclusions (10, 11). Mendelian randomization (MR), an analytical approach using genetic variation, allows researchers to use tools such as single nucleotide polymorphisms (SNPs) to simulate the random allocation found in randomized controlled trials, helping to uncover potential causal relationships (12). This method is particularly valuable in situations where long-term randomized controlled trials are impractical or where issues of confounding and reverse causation need to be addressed (13). In recent years, MR has been widely used as a reliable method in genetic epidemiology for investigating the causal relationships between modifiable exposures and clinical outcomes (14, 15).

This study aims to investigate the potential causal relationship between physical activity, sedentary behavior, and the risk of sepsis using a two-sample MR analysis based on genome-wide association study (GWAS) data.

## Materials and methods

### Study design

This study adopts the MR approach using summary statistics derived from GWAS to investigate the relationship between physical activity, sedentary behavior, and the risk of sepsis occurrence. This study adheres to the three core assumptions of MR analysis: (1) the instrumental variables are strongly associated with the exposure factors (physical activity and sedentary behavior); (2) the instrumental variables are independent of any known confounders; (3) the instrumental variables influence the outcome (risk of sepsis) exclusively through pathways associated with the exposure factors (16) (Supplementary Figure 1).

### Data sources

Physical activity data were obtained from the UK Biobank (UKB) GWAS database. The UKB is a large-scale prospective cohort study involving approximately 500,000 adults from 22 centers across the United Kingdom. This study includes three types of physical activity: self-reported moderate to vigorous physical activity (MVPA), self-reported vigorous physical activity (VPA), and accelerometer-based physical activity. Self-reported physical activity data were collected using a touchscreen questionnaire similar to the international physical activity questionnaire (17). This methodology categorizes different forms of exercise into moderate-intensity and vigorous-intensity activities and calculates the time spent in these activities. MVPA is calculated by multiplying the minutes of moderate activity by 4 and adding the minutes of vigorous activity multiplied by 8, where 4 and 8 represent metabolic equivalents. VPA is categorized into two groups: individuals reporting 0 days of VPA per week and those reporting more than 3 days per week with at least 25 min of VPA per day. The accelerometer-based physical activity data were collected using the wrist-worn Axivity AX3 accelerometer, which was worn by participants for a minimum of 3 days and a maximum of 7 days, resulting in an average acceleration value (milli-gravity, mg). The questionnaire content, data cleaning, and other details of the physical activity phenotype described above can be found in the original study by Klimentidis et al. (18).

Data on sedentary behavior were also obtained from the UKB database and included three types of sedentary behavior: television watching, computer use, and driving. Data collection was based on participants’ responses to three questions about their daily television viewing time, non-work-related computer time, and daily driving time. The questionnaire content, data cleaning, and other details can be found in the original study by van de Vegte et al. (15).

GWAS data for sepsis were obtained from the FinnGen database, including 10,666 sepsis cases and 303,314 controls (19). In the FinnGen database, sepsis is defined by International Classification of Diseases, 10th Revision (ICD-10) code A41 for patient discharge or mortality, and by 8th Revision (ICD-8) and 9th Revision (ICD-9) codes 038. Detailed information on all the included GWAS can be found in Supplementary Table 1.

The GWAS data from the UKB and FinnGen databases were ethically approved by their respective institutions. Informed consent was obtained from all participants in the contributing studies, and the data used in this study are publicly available and freely accessible.

### Statistical analysis

According to the three assumptions of MR analysis, SNPs selected as instrumental variables had to meet the following criteria: genome-wide significance level (*P* < 5e–8), no linkage disequilibrium (*r*^2^ < 0.001 and kb = 10,000), and all instrumental variables with an F-statistic value greater than 10.

Three methods of MR analysis were used to assess the potential causal effect of physical activity and sedentary behavior on sepsis: Inverse Variance Weighted (IVW), Weighted Median, and MR-Egger, with the results expressed as odds ratios (OR) and 95% confidence intervals (95% CI). The IVW method, which combines the Wald ratio estimates of each SNP to obtain an overall causal estimate, served as the primary analytical approach in this study. The Weighted Median and MR-Egger methods were used to validate and strengthen the robustness of the IVW results.

Cochran’s *Q* test was used to identify heterogeneity among individual SNP estimates. The MR-Egger intercept test was used to assess horizontal pleiotropy, with *P* < 0.05 indicating potential horizontal pleiotropy. The leave-one-out approach was used to determine whether outcomes were significantly influenced by a single SNP.

All statistical analyses were performed using R 4.2.3 and the “TwoSampleMR” package.

## Results

### MR assessment of the causal effect of physical activity and sedentary behavior on sepsis risk

**FIGURE 1:**
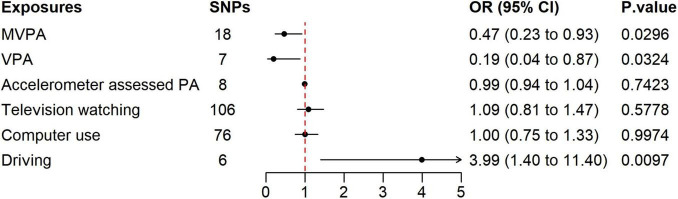
Forest plot of the association between physical activity, sedentary behavior, and sepsis using the IVW method. SNP, single nucleotide polymorphism; MVPA, moderate to vigorous physical activity; VPA, vigorous physical activity; PA, physical activity.

Figure 1 shows the results of the IVW analysis correlating exposure factors with sepsis, which demonstrated a significant association between the genetically predicted duration of MVPA and reduced sepsis risk (OR 0.47, 95% CI 0.24–0.93, *P* = 0.0296). Similarly, the genetically predicted duration of VPA was associated with a reduced risk of sepsis (OR 0.19, 95% CI 0.04–0.87, *P* = 0.0324). However, no significant association was observed between accelerometer assessed physical activity and sepsis risk.

Daily duration of computer use and television watching showed no significant association with sepsis risk. In contrast, the genetically predicted duration of daily driving was significantly associated with an increased risk of sepsis (OR 3.99, 95% CI 1.40–11.40, *P* = 0.0097). Detailed results of the MR analysis are shown in Supplementary Tables 2, 3 and Supplementary Figure 2.

### Heterogeneity, horizontal pleiotropy, and sensitivity analysis

Heterogeneity testing revealed evidence of heterogeneity in the analysis of the association between MVPA and sepsis risk based on the Cochran Q test, resulting in penalized weights in the IVW method. Potential heterogeneity was also noted in the analysis of television watching and sepsis risk (Table 1).

**TABLE 1:** Results of Cochrane’s *Q* test and pleiotropy test.

Exposures	Cochrane’s *Q* test		Pleiotropy test		
	**Q**	***P*-value**	**Egger intercep**	**SE**	***P*-value**
MVPA	30.3	0.0164	0.0328	0.0289	0.2729
VPA	8.3	0.1401	–0.0413	0.0573	0.5032
Accelerometer assessed PA	6.1	0.4173	–0.0357	0.0295	0.2710
Television watching	135.6	0.0204	0.0020	0.0087	0.8165
Computer use	76.7	0.3931	–0.0097	0.0104	0.3529
Driving	3.3	0.4977	0.0829	0.0621	0.2529

MVPA, moderate to vigorous physical activity; VPA, vigorous physical activity; PA, physical activity.

MR-Egger intercept tests indicated the absence of horizontal pleiotropy in all analyses (Table 1). Leave-one-out sensitivity analysis showed that the causal relationships between MVPA, VPA, and driving time on sepsis risk were not biased by individual SNPs, confirming the results as clear and reliable (Supplementary Figures 3–5).

## Discussion

In this study, we used large-scale GWAS summary data to explore the potential causal relationships between physical activity, sedentary behavior, and the risk of sepsis. The results indicate that genetically predicted MVPA and VPA are significantly inversely associated with the risk of sepsis, whereas prolonged driving is significantly associated with an increased risk of sepsis. Given that physical activity and sedentary behavior are modifiable risk factors, these findings may offer new perspectives for the prevention of sepsis and suggest potential intervention methods for the development of personalized prevention strategies.

Physical activity is widely recognized as beneficial for health, reducing the risk of diabetes, cardiovascular disease, and stroke (6, 20, 21). Research in animal models has shown that moderate-intensity exercise improves survival in rats with sepsis, possibly by suppressing inflammatory responses, reducing oxidative and nitrosative stress, and activating antioxidant defense mechanisms (22). Human studies also indicate that physical exercise can effectively inhibit endotoxin-induced TNF-α production (23). An epidemiologic study of American walkers and runners suggested that physical inactivity may increase the risk of mortality from sepsis (10).

Our study also found that MVPA and VPA were associated with a reduced risk of sepsis, with VPA showing a more pronounced protective effect compared to MVPA. This finding aligns with a recent large cohort study, which observed that individuals engaging in 1 h of exercise per week had a significantly lower risk of infection or sepsis compared to those exercising less than 1 h per week, with the risk decreasing further as exercise duration increased (11). However, it is noteworthy that some studies have reported that intense exercise, such as that performed by professional athletes, may increase the risk of infection (24). In addition, our study found no significant association between accelerometer-assessed physical activity and the risk of sepsis. This discrepancy may be due to conceptual differences between accelerometer-assessed physical activity and self-reported physical activity (25). Several studies have shown significant differences between these two methods of measuring physical activity (25–27). The choice of measurement method can significantly impact the assessment of physical activity, and notable differences have been observed in MR studies regarding the relationship between accelerometer-assessed physical activity and self-reported physical activity with various outcomes (28, 29).

There is substantial evidence that sedentary behavior is associated with several health problems, including increased incidence and mortality risk of obesity, diabetes, cardiovascular disease, and cancer (5, 30). However, studies on the relationship between sedentary behavior and sepsis are less frequent. In a community-based study, the highest incidence of sepsis was found in those with low levels of physical activity and who watched television for ≥ 4 h/day. After adjusting for confounding factors, a low level of physical activity was independently associated with an increased incidence of sepsis, while television watching showed no significant correlation with the incidence of sepsis (31). There have been no prior studies reported on the relationship between computer use and driving with the risk of sepsis. In our study, only driving time was found to be associated with the risk of sepsis, while watching television and using a computer did not show a significant correlation with sepsis risk. This may be due to the biological heterogeneity among different sedentary behaviors. In the study by van de Vegte et al. the genetic pathways related to television watching and computer use involved genes associated with the nervous system, whereas the genetic pathways related to driving did not (15). Notably, significant differences have also been observed in MR studies involving other diseases, concerning these three sedentary behaviors (15, 32). Furthermore, in our study, the confidence intervals for the relationship between driving and sepsis were relatively wide, reflecting uncertainty in the estimates. This underscores the need for further research to validate our findings and explore the underlying mechanisms.

This study also had several limitations. First, the GWAS data included only European populations, which may limit the generalizability of our findings across races. Second, the study did not distinguish between different types of physical activity, such as swimming or running. The effects of these different types of physical activity on susceptibility to sepsis may also differ. Furthermore, although our study identified potential causal relationships between physical activity, sedentary behavior, and sepsis risk, the underlying biological mechanisms require further investigation. Finally, although the MR approach offers significant advantages in controlling for confounders, sepsis is a complex disease with multiple independent or interacting biological pathways. The MR method may not fully capture the effects of these complex factors. Future research should consider stratified analyses by age, sex, or comorbid conditions to gain a more comprehensive understanding of exposure-outcome relationships in different populations.

## Conclusion

This study provides preliminary evidence of a causal relationship between MVPA and VPA and a reduced risk of sepsis, while prolonged sedentary behaviors such as driving are positively associated with an increased risk of sepsis. These findings highlight the importance of addressing physical activity and sedentary behavior in sepsis prevention strategies.

## Data Availability

Publicly available datasets were analyzed in this study. This data can be found here: https://gwas.mrcieu.ac.uk/ and https://r8.finngen.fi/.
