# Supplementary material for: Physical activity, sedentary behavior, and risk of sepsis: a two-sample mendelian randomization study
Source: Front Med (Lausanne). 2024 Aug 19;11:1436546. doi: 10.3389/fmed.2024.1436546 (PMC11366612; doi:10.3389/fmed.2024.1436546)
Supplement: Supplementary file 1 [file Data_Sheet_1.pdf]

Physical Activity, Sedentary Behavior, and Risk of Sepsis: A Two-Sample Mendelian Randomization Study

Yang Zhang [1,2], Yu Rong[1,3], Jun Mao[1,3], Jin Zhang[1, 2], Wenyan Xiao[1, 2], Min Yang [1, 2*]
[1] The Second Department of Critical Care Medicine, The Second Affiliated Hospital of Anhui Medical University, Hefei, Anhui 230601, P. R. China
[2] Laboratory of Cardiopulmonary Resuscitation and Critical Care, The Second Affiliated Hospital of Anhui Medical University, Hefei, Anhui 230601, P. R. China
[3] Anqing Municipal Hospital, Anqing, Anhui 246003, P. R. China
*Correspondence: Min Yang, email: yangmin@ahmu.edu.cn

Table 1 Details of the GWASs included in the Mendelian randomization

Traits	Data source	Participants	Ancestry	Pubmed ID
MVPA	UK Biobank	377,234	European	29899525
VPA	UK Biobank	261,055	European	29899525
Accelerometer assessed PA	UK Biobank	91,084	European	29899525
Television watching	UK Biobank	437,887	European	32317632
Computer use	UK Biobank	360,895	European	32317632
Driving	UK Biobank	310,555	European	32317632
Sepsis	FinnGen	313,980	European	36653562

MVPA moderate to vigorous physical activity, *VPA* vigorous physical activity, *PA* physical activity

Table 2 Mendelian Randomization estimates between physical activity and sepsis risk

Exposures	Methods	SNPs	OR (95%CI)	P value
**MVPA**				
	MR Egger	18	0.0513 (0.0011, 2.4641)	0.1522
	WM	18	0.3892 (0.1866, 0.8120)	0.0119
	IVW	18	0.4668 (0.2350, 0.9274)	0.0296
**VPA**				
	MR Egger	7	15.8023 (0.0001, 2698997)	0.6722
	WM	7	0.2217 (0.0449, 1.1045)	0.0660
	IVW	7	0.1949 (0.0436, 0.8717)	0.0324
**Accelerometer assessed PA**				
	MR Egger	8	1.1367 (0.9060, 1.4261)	0.3108
	WM	8	1.0184 (0.9537, 1.0875)	0.5861
	IVW	8	0.9913 (0.9412, 1.0441)	0.7423

MVPA moderate to vigorous physical activity, *VPA* vigorous physical activity, *PA* physical activity, *IVW* inverse variance weighted, *WM* weighted median, *SNP* single nucleotide polymorphism, *OR* odds ratio, *CI* confidence interval.

Table 3 Mendelian Randomization estimates between Sedentary Behavior and sepsis risk

Exposures	Methods	SNPs	OR (95%CI)	P value
**Television watching**				
	MR Egger	106	0.9159 (0.2059, 4.0749)	0.9084
	WM	106	1.0810 (0.7236, 1.6148)	0.7038
	IVW	106	1.0894 (0.8059, 1.4726)	0.5778
**Computer use**				
	MR Egger	76	2.1389 (0.4229, 10.8183)	0.3609
	WM	76	1.1575 (0.7493, 1.7879)	0.5098
	IVW	76	0.9995 (0.7485, 1.3347)	0.9974
**Driving**				
	MR Egger	6	0.0046 (0.0001, 100.1359)	0.3507
	WM	6	4.1093 (1.0645, 15.8635)	0.0403
	IVW	6	3.9916 (1.3982, 11.3958)	0.0097

IVW inverse variance weighted, *WM* weighted median, *SNP* single nucleotide polymorphism, *OR* odds ratio, *CI* confidence interval.

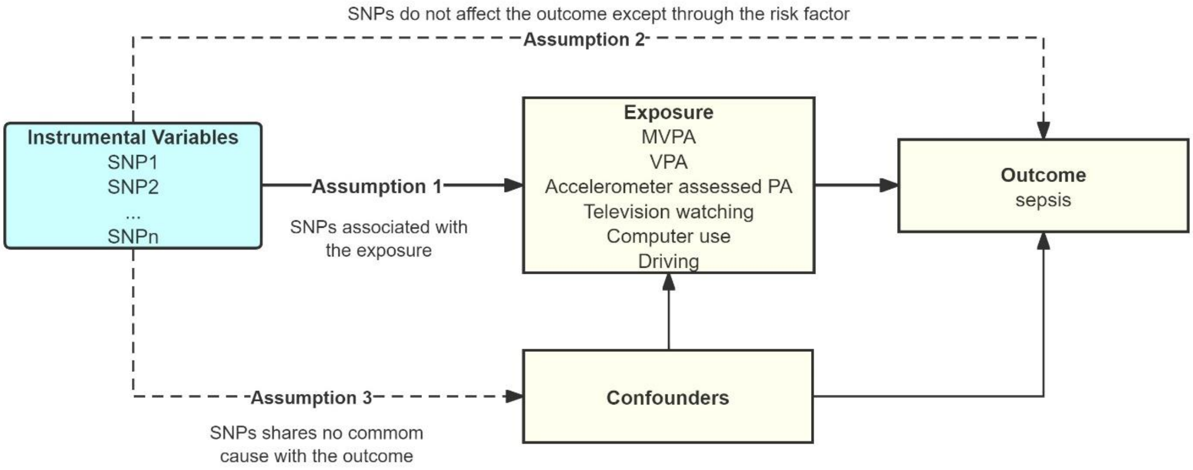

Figure 1 The overview of the study design.

SNP single nucleotide polymorphism, *MVPA* moderate to vigorous physical activity, *VPA* vigorous physical activity, *PA* physical activity

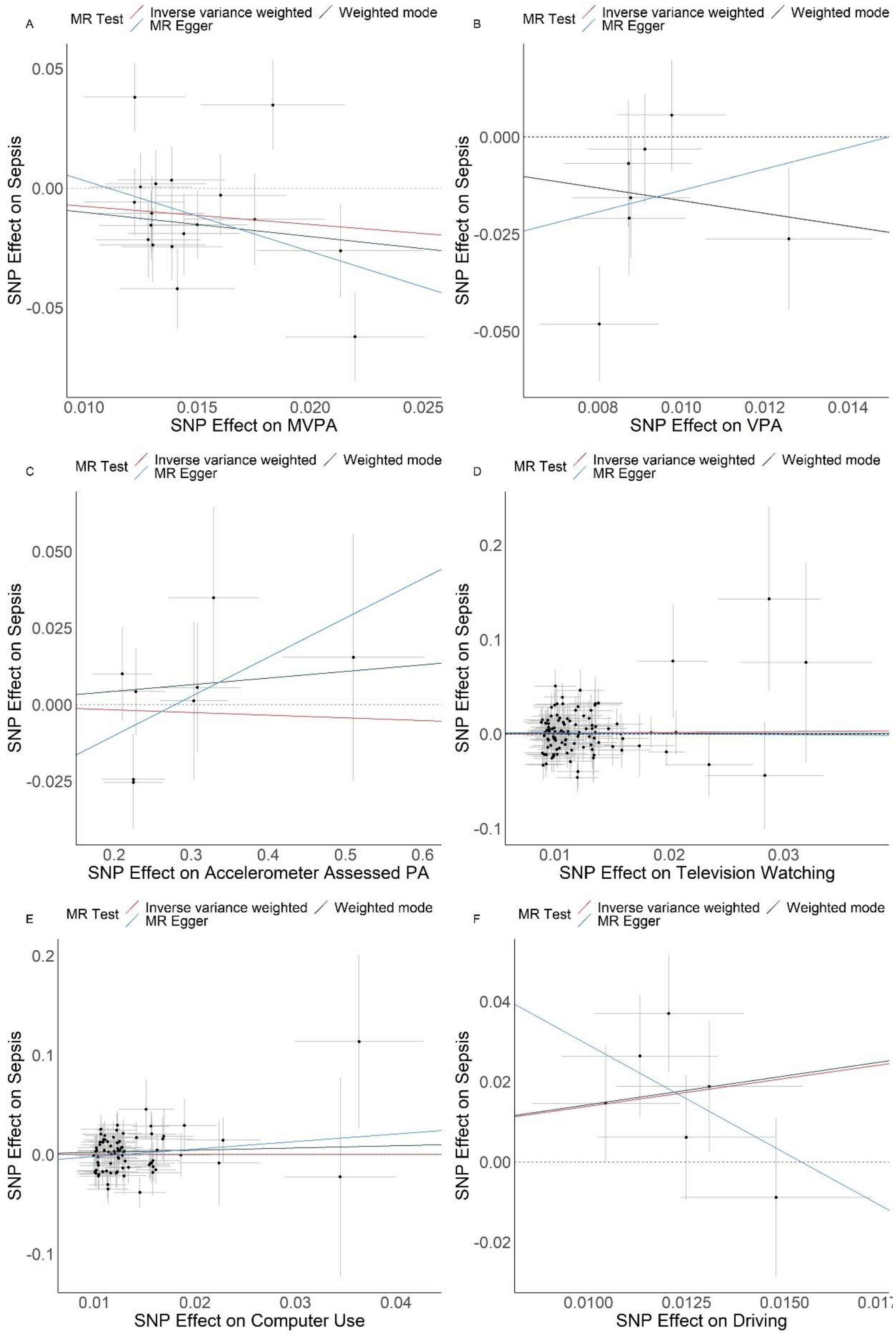

Figure 2 Scatter plots of results from Mendelian randomization analysis.

SNP single nucleotide polymorphism, *MVPA* moderate to vigorous physical activity, *VPA* vigorous physical activity, *PA* physical activity

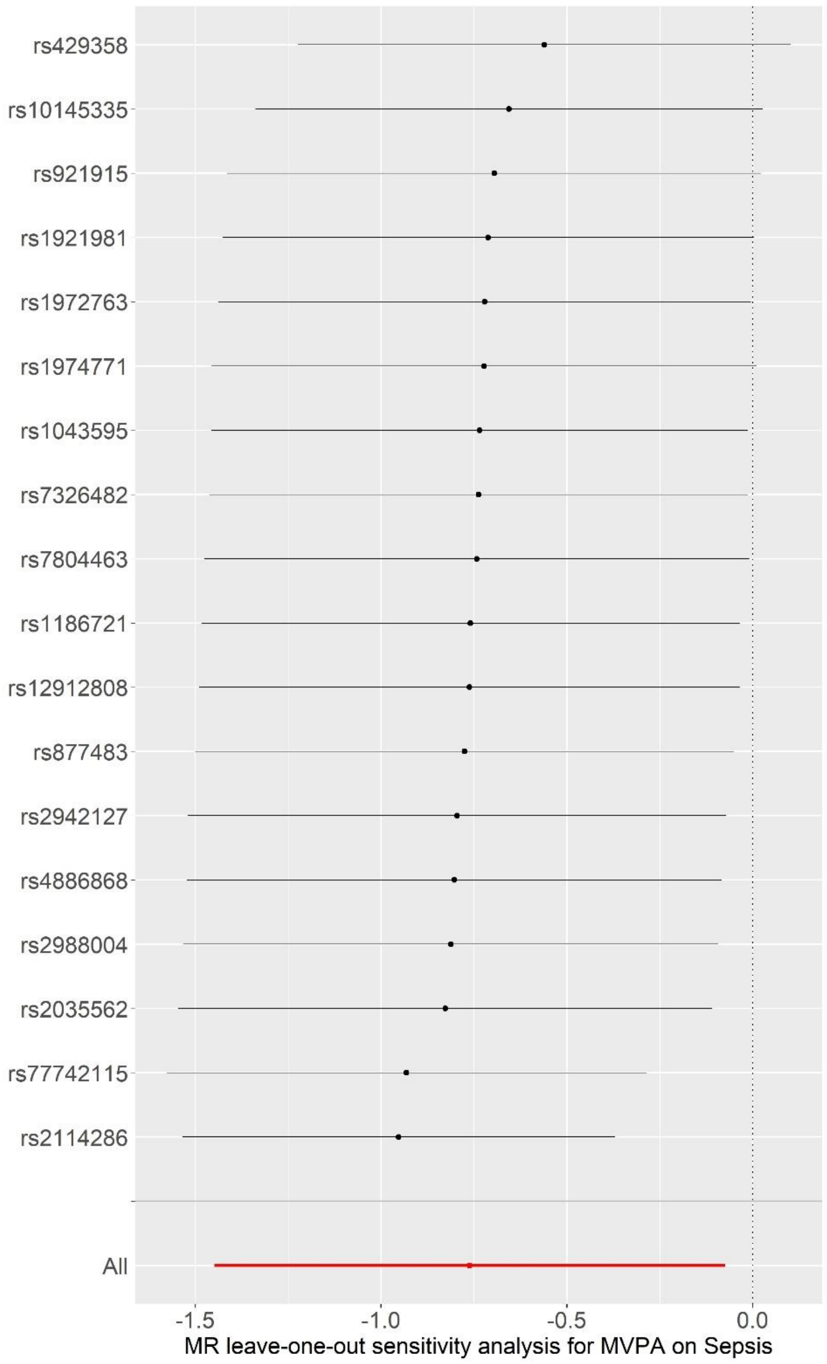

Figure 3 Leave-one-out plots for the causal association between MVPA and sepsis

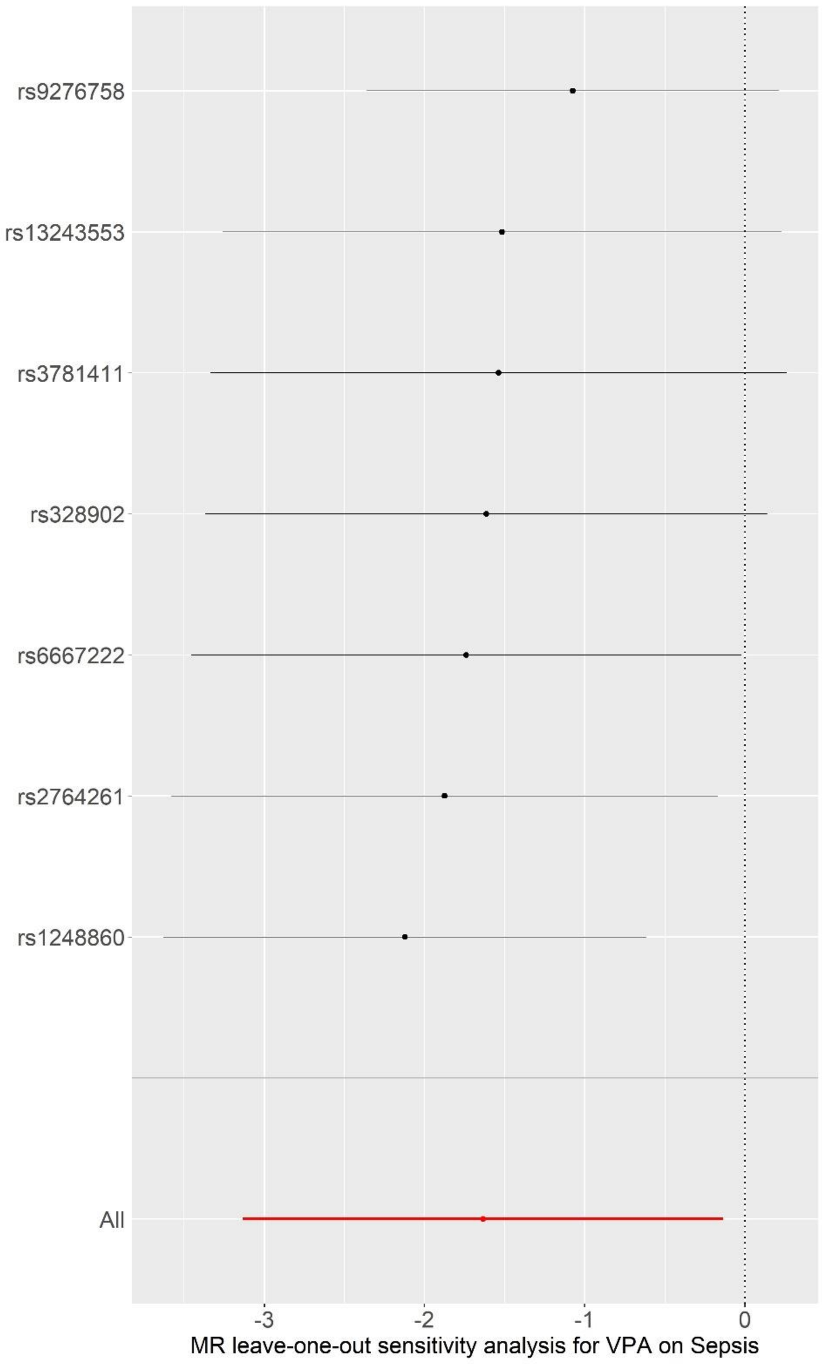

Figure 4 Leave-one-out plots for the causal association between VPA and sepsis

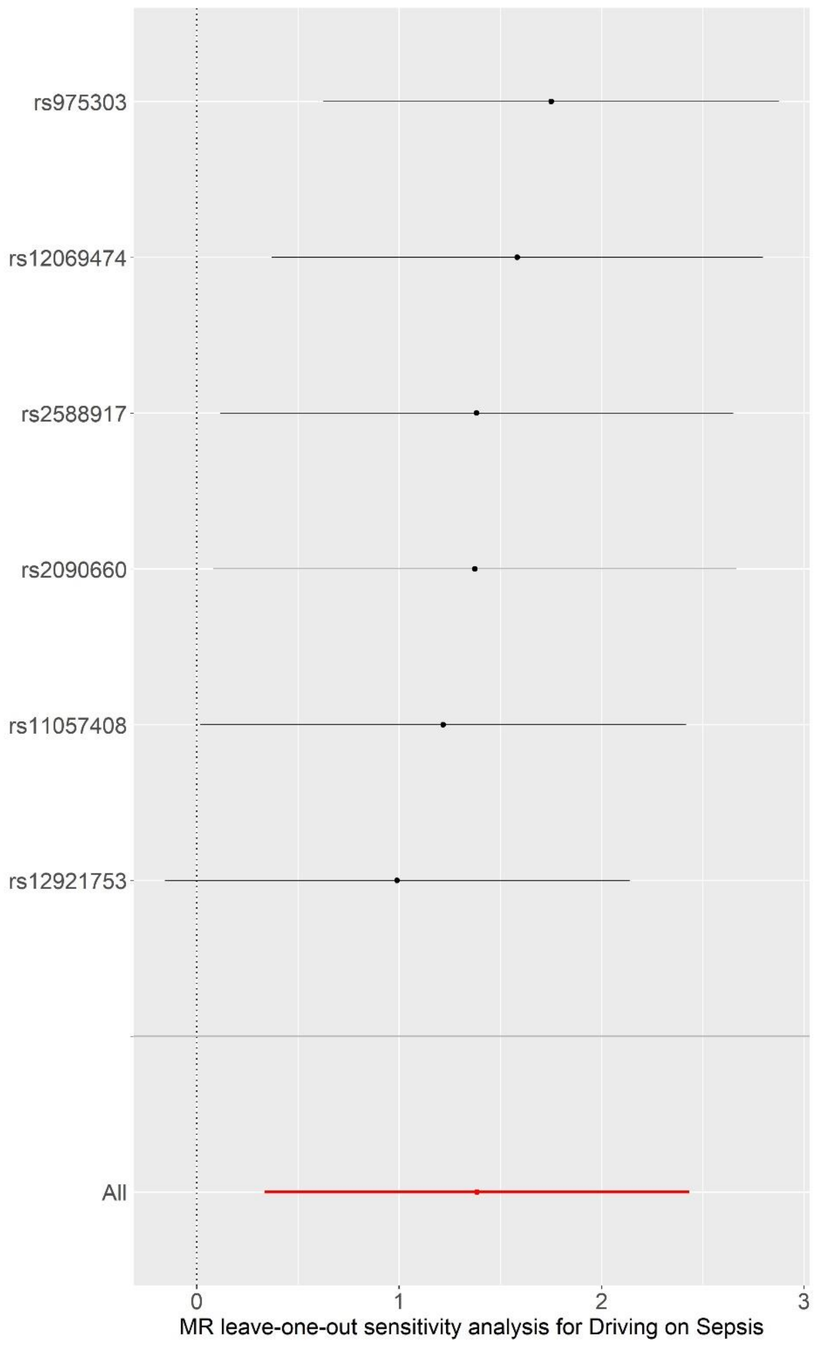

Figure 5 Leave-one-out plots for the causal association between driving and sepsis
